# Learning From the Exceptions: HIV Remission in Post-treatment Controllers

**DOI:** 10.3389/fimmu.2019.01749

**Published:** 2019-07-24

**Authors:** Behzad Etemad, Elmira Esmaeilzadeh, Jonathan Z. Li

**Affiliations:** Harvard Medical School, Brigham and Women's Hospital, Boston, MA, United States

**Keywords:** HIV, post-treatment controllers, remission, treatment interruption, elite controllers

## Abstract

Among the top priorities of the HIV field is the search for therapeutic interventions that can lead to sustained antiretroviral therapy (ART)-free HIV remission. Although the majority of HIV-infected persons will experience rapid viral rebound after ART interruption, there are rare individuals, termed post-treatment controllers (PTCs), who demonstrate sustained virologic suppression for months or years after treatment cessation. These individuals are considered an ideal example of durable HIV control, with direct implications for HIV cure research. However, understanding of the mechanisms behind the capacity of PTCs to control HIV remains incomplete. This is in part due to the scarcity of PTCs identified through any one research center or clinical trial, and in part because of the limited scope of studies that have been performed in these remarkable individuals. In this review, we summarize the results of both clinical and basic research studies of PTCs to date, explore key differences between PTCs and HIV spontaneous controllers, examine potential mechanisms of post-treatment control, and discuss unanswered questions and future research directions in this field.

## Introduction

Within each medical field, there exist individuals who exhibit extreme responses to medical treatment. As an example, individuals who have an unexpectedly dramatic response to cancer therapy are termed “exceptional responders.” These exceptional responders represent an area of intense research interest within the oncology field (1) and have already made important contributions to the understanding of both basic tumor biology and drug development (2). In this review, we focus on a group of exceptional responders within the HIV field, specifically individuals who were treated with antiretroviral therapy (ART) and can subsequently maintain HIV remission even when the ART is discontinued.

**Figure 1:**
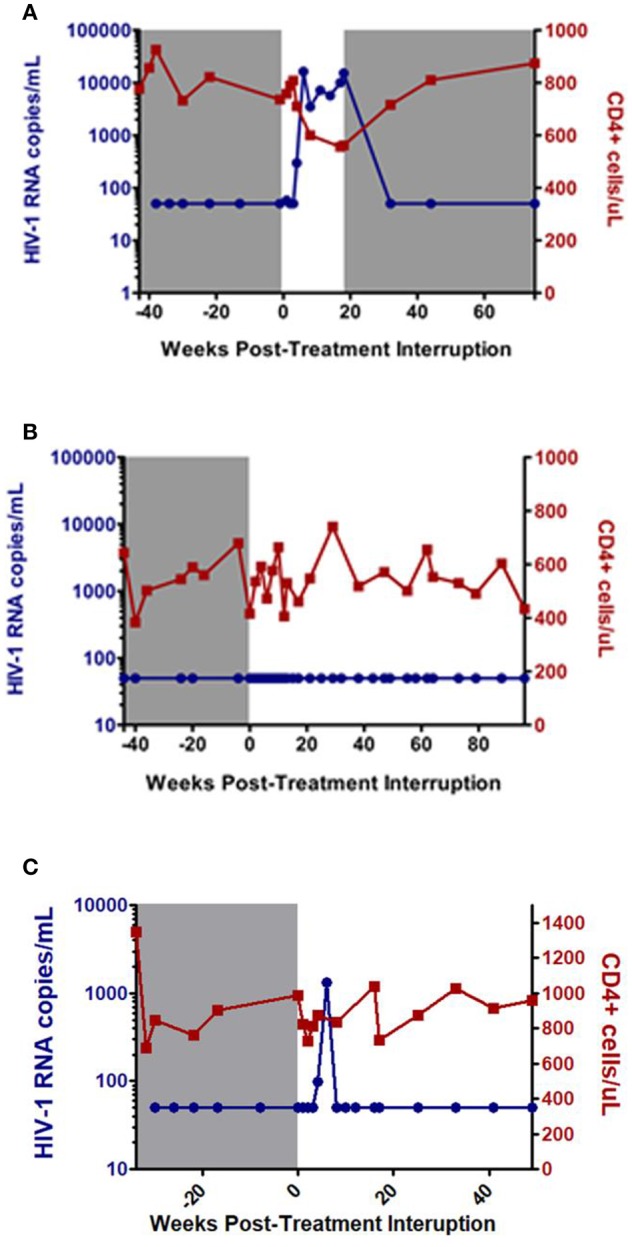
Examples of post-treatment non-controller (NC) **(A)**, and post-treatment controllers **(B,C)**. Gray shaded area represents time on antiretroviral therapy. Adapted from CHAMP study (6).

HIV infection is characterized by sustained viral replication and progressive decline in CD4 cell counts (3). ART is effective in suppressing viral replication and decreasing HIV-associated morbidity and mortality, but it cannot completely eradicate all HIV-infected cells. Consequently, HIV viral load rebounds rapidly after treatment interruption in most HIV patients (4, 5). However, there are rare individuals, termed post-treatment controllers (PTCs), who are able to suppress the virus for a prolonged period of time after treatment interruption (Figure 1). These individuals are considered an ideal example of durable HIV control and have the potential to provide substantial insight into the “natural” mechanisms of functional cure and sustained HIV remission (7).

Interest in ways to induce post-treatment control were initially kindled by a report of an individual who was able to control HIV without ART after undergoing several sequential treatment interruptions (8) and in an in-depth report of 14 early-treated PTCs reported in the VISCONTI study (7). There have been a number of subsequent studies of PTCs with a wide range of reported frequency amongst those who discontinue ART (6, 7, 9–19). This variation in reported frequency of PTCs may be attributed to different baseline characteristics of the populations in which these studies were done, as well as the heterogeneous definitions applied for defining this rare group of HIV patients (18). In this review, we will summarize the most recent findings on the clinical and immunological characteristics of PTCs, differentiate them from HIV spontaneous controllers (SCs), and discuss the role of PTCs in the search for strategies toward HIV remission and cure.

## Post-Treatment Controller Definitions

Since the initial description of the post-treatment controller phenotype, a number of observational studies and interventional clinical trials have been performed to investigate the characteristics of this rare group of patients and to determine the mediators of post-treatment control. However, the heterogeneities in study designs have made it challenging to compare studies and to gain a clear grasp of the PTC population. For example, the definition of post-treatment control has differed dramatically between studies. Some studies have considered virologic rebound to be a plasma viral load above 50 HIV-1 RNA copies/ml after treatment interruption, while others have used a threshold of 400 HIV-1 RNA copies/ml or 1,000 HIV-1 RNA copies/ml for this purpose (Table 1, Supplementary Table S1). The duration of viral control after treatment interruption has also differed dramatically between studies and ranged from a median of 6 month to more than 2 years (7, 9–32). Furthermore, the loss of viral control was also defined differently between previous studies. Some considered 2 consecutive viral loads above 50 HIV-1 RNA copies/ml to indicate the loss of post-treatment control (8–10), while others considered 1–4 consecutive viral loads higher than 400 HIV-1 RNA copies/ml as the definition for viral rebound post-treatment interruption (7, 12, 16–18). Of note, the largest PTC study to date has been the Control of HIV after Antiretroviral Medication Pause (CHAMP) study, which identified 67 PTCs through the pooled analysis of 14 clinical studies from the AIDS Clinical Trials Group (ACTG) and other North American cohorts (6, 14, 20–32). In this study, the PTCs were defined as individuals who maintained viral loads ≤ 400 copies/mL at two-thirds or more of time points for ≥24 weeks post treatment interruption (6).

**Table 1:** Post-treatment controller (PTC) frequency after treatment interruption reported from previously published studies.

**References**	**Cohort**	**Timing of ART**	**PTC, Total, *N***	**PTC Frequency (%)**	**VF threshold copies/ml**	**PTC duration**
Hocqueloux et al. (9)	ANRS	Early	5	15.6	>50	75 months (median)
Goujard et al. (10)	ANRS PRIMO	Early	14	8.5	>50	4.5 years (median)
Lodi et al. (11)	CASCADE	Early	11	5.5	>50	24 months
Saez-Cirion et al. (7)	VISCONTI	Early	14	15.3	>400	89 months (median)
Stohr et al. (12)	SPARTAC	Early	4	2.4	>400	164–202 weeks
Van Gulck et al. (15)	Secondary Controllers	Chronic	4		>1,000	At least 6 months
Assoumou et al. (16)	ANRS SALTO	Chronic	7	4.2	>400	12 months (7 patients) 36 months (4 of the 7 patients)
Calin et al. (17)	ULTRASTOP	Early Chronic	1	10	>400	56 weeks
Perkins et al. (18)	NHS	Chronic	4	4.2	>400	267–1,058 days
Fidler et al. (19)	CASCADE	Early	22	2.8	>50	24 months
Namazi et al. (6)*	CHAMP	Early & Chronic	67	13 (Early) 4 (Chronic)	>400	24–804 weeks

* *The CHAMP study includes participants from 8 AIDS Clinical Trials Group (ACTG) studies [ACTG 371 (20), A5024 (21), A5068 (22), A5102 (23), A5130 (24), A5170 (25), A5187 (26), and A5197 (27)], the Montreal Primary HIV Infection Cohort (Montreal PIC) (28), the Seattle Primary Infection Program (SeaPIP) (13, 29), the University of California San Diego Primary Infection Cohort (UCSD PIC) (14), a National Institutes of Health (NIH) therapeutic vaccine trial (30), the University of California San Francisco (UCSF) OPTIONS study (31), and the Ragon HIV Controllers cohort (32)*.

*ART, Anti Retroviral Therapy; VF, Viral Failure*.

## Demographic Characteristics of PTCs

The median age of PTCs in these studies ranged from 27 to 46 years old. The majority of PTCs identified were male, likely reflecting the sex distribution of the clinical trial participants (6, 7, 9, 11, 12, 15–18). Intriguingly, there have been reports that female gender may be associated with a higher chance of post-treatment HIV control (10) and spontaneous control (33, 34), highlighting the need for studies focusing on female participants of treatment interruption trials. In addition, the majority of PTCs have been reported by studies from North America and Europe (6, 7, 9–12, 15–18) and little is known about PTCs from outside of those regions. In an analysis of SPARTAC trial participants who initiated ART during early HIV infection, individuals with delayed viral rebound could be identified from participants enrolled in South Africa and Uganda (35). Furthermore, African participants tended to have lower pre-ART viral load and integrated HIV DNA levels, and after treatment interruption, Africans appeared to experience a longer duration of viral remission than non-Africans in the SPARTAC study (12, 36). These results provide a strong rationale for additional studies of PTCs from Africa and other regions to assess the impact of race and HIV subtype on barriers to HIV remission.

## Clinical and Immunological Characteristics

**Figure 2:**
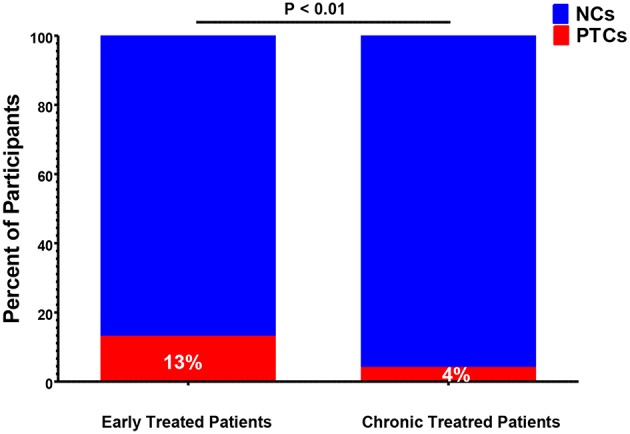
Frequency of post-treatment controllers (PTCs) identified in early-vs. chronic-treated participants of the CHAMP study (6). NCs, post-treatment non-controllers.

Historically, the majority of PTCs have been identified in studies of patients who initiated ART during early HIV infection (7, 9–12, 20, 26, 29–31, 37). However, PTCs have also been identified in participants who were treated during chronic HIV infection (15, 16, 18, 21, 22, 25, 27, 38). The CHAMP study directly compared the frequency of post-treatment control between individuals who initiated ART during early and chronic HIV infection. This study found that individuals who were treated during early infection were far more likely to meet the PTC criteria after treatment interruption compared to those treated during chronic infection (13 vs. 4%, *P* < 0.01, Figure 2) (6).

At the time of treatment interruption, CD4 cell counts for the PTCs were generally quite high with a median of 720 to 1,429 cells/mm^3^ amongst the studies (7, 9–12, 15–19). After ART discontinuation, PTCs can exhibit a range of viral load dynamics with a subset demonstrating persistent viral load suppression (Figure 1B) while others experience early viral rebound before subsequently regaining viral control (Figure 1C). In the CHAMP study, ~45% of PTCs had early viral load peaks ≥1,000 HIV-1 RNA copies/mL and 33% had early viral load peaks ≥10,000 HIV-1 RNA copies/mL amongst those with intensive weekly viral load monitoring (6).

The comparison of previously published PTC studies has also been difficult due to heterogeneity in the inclusion of PTCs with varying duration of viral control. To place the PTC studies in context, the median time of HIV rebound after ART interruption for post-treatment non-controllers (NCs) is ~3–4 weeks and only a small proportion of non-controllers are able to maintain viral suppression to 12 weeks or beyond (4). The VISCONTI study was one of the earliest and most comprehensive of the PTC studies (7). The inclusion criteria for the 14 VISCONTI participants were individuals who were treated during early HIV infection and maintained viral suppression <400 HIV-1 RNA copies/mL for at least 2 years after ART interruption. To assess the durability of HIV remission, the CHAMP study used a more inclusive definition of post-treatment control (viral suppression for 24 weeks). In this analysis, the median duration of post-treatment control was a little over 2 years and the proportion of PTCs who remained virologically suppressed in years 1–5 were 75, 55, 41, 30, and 22%, respectively (6). These results show that post-treatment control is not always durable and that PTCs will require continued clinical and virologic monitoring. These results highlight the heterogeneity in the post-treatment controller phenotype, with some individuals losing control within 1 year and others maintaining viral suppression for more than 10 years (6, 7). While the latter group may be the best model of sustained HIV remission, uncovering factors that lead to the loss of viral control in PTCs may also provide insight on the mechanisms behind their HIV remission. It should be noted though, that the rates of viral suppression reported in the PTCs are in the absence of any additional interventions and that strategies to augment key HIV-specific immune responses have the potential to improve the durability of post-treatment control.

## Comparing Spontaneous and Post-Treatment Controllers

Without ART, most HIV-infected individuals will have high levels of HIV-1 RNA and experience progressive absolute CD4+ T-cell decline, clinical immunodeficiency, and death (39). However, a small proportion of those infected with HIV can spontaneously maintain very low levels of plasma viral load without the use of antiretroviral therapy (ART) (40, 41). The existence of these HIV spontaneous controllers (SCs), also known as elite controllers (ECs), represented the first indication that the goal of drug-free HIV remission is possible. Although these SCs have low or even undetectable viremia by conventional viral load assays, they generally harbor replication competent virus and have evidence of ongoing viral replication and evolution (42–45). Through robust genetic and functional studies, the most consistent mediator of spontaneous HIV control appears to be through the effects of cytotoxic CD8 T lymphocyte (CTL) responses (46, 47), and the protective effects of certain HLA alleles, such as HLA B^*^27 and B^*^57 (48–50). Similar to the PTCs, SCs appear to be a heterogeneous population of individuals with respect to the level and durability of HIV control (51, 52). While some SCs can maintain viral loads <50 copies/ml in absence of ART (i.e., elite controllers [ECs]) (41, 53). Viremic controllers (VCs) can maintain a less robust level of viral suppression, with detectable viral loads below 2,000 HIV-1 RNA copies/mL in the absence of ART (54).

However, even amongst the ECs, there is evidence of heterogeneity in immune responses (49, 55), and a subset will lose viral control and experience immunological and clinical progression over time (56–58). Low Gag-specific CD8 T cell response, high levels of inflammatory cytokines and high viral diversity have been reported as factors that predict loss of viral control in ECs (51).

Due to the rarity of individuals undergoing treatment interruption, PTCs have for a long time not been recognized as a separate entity from SCs. While it is possible that some PTCs treated during early HIV infection may have achieved spontaneous control in the absence of ART, there are now several lines of evidence that PTCs are indeed distinct from HIV SCs: (1) CTL responses have been found to be far weaker in PTCs compared to SCs (7); (2) Unlike SCs, PTCs do not appear to be enriched in protective HLA alleles (3, 10, 59), with the VISCONTI study reporting a high frequency of HLA alleles previously associated with less favorable clinical outcomes (7); (3) PTCs frequently present with symptomatic acute retroviral syndrome and have pre-ART viral loads that are similar to that of non-controllers, but significantly higher than that of HIV SCs (6, 7); and (4) Results from both the SPARTAC and CHAMP studies have demonstrated an ART-specific effect as early ART initiation significantly increases the chances of achieving post-treatment control (6, 35). Together, these findings support the concept that PTCs are largely distinct from SCs and represent individuals who would not have been able to achieve HIV remission without the period ART.

## Mechanisms and Predictors of Post-Treatment Controllers

While the exact mechanism behind the ability of PTCs to maintain HIV remission remains unclear, there is evidence for an unusual degree of reservoir restriction and relatively weak HIV-specific CTL activity. In prior studies of ART-treated individuals, the HIV reservoir is primarily maintained within memory CD4 T cells, especially those of central memory (T_CM_) and transitional memory (T_TM_) cells (60). In prior treatment interruption studies, smaller total and active HIV reservoirs before treatment interruption have been associated with delayed HIV rebound after treatment interruption. Specifically, lower levels of pre-treatment interruption HIV proviral DNA have predicted delayed viral rebound (16, 61), as has lower levels of cell-associated HIV RNA (4, 30). In PTCs, levels of HIV DNA and cell-associated RNA have also been found to be low in some studies (10, 15) but not others (38). In the VISCONTI analysis, the predominant cellular subset contributing to the HIV reservoir has been reported to be the T_TM_ cells (7), similar to that found in other early treated patients (62) and suggest that the low frequency of HIV infection within the longest-lived CD4 T cells (naïve and central memory) may contribute to post-treatment control. In studies of SCs, there have been reports that the HIV reservoir is also restricted within the T_CM_ cell subset (63), although this has not been replicated in other studies (7). In ART-treated individuals, the vast majority of HIV proviral DNA are defective and until recently, the proviral landscape within PTCs had not been investigated. In an analysis of ACTG PTCs, Sharaf et al. reported near-full length proviral sequencing results showing that PTCs had an ~7-fold smaller HIV reservoir compared to NCs prior to the ATI, but that some PTCs had relatively large fractions of intact proviruses (64). In a separate case report, post-treatment control could be maintained despite the presence of a clonally-expanded population of HIV-infected cells harboring replication-competent virus (65). Overall, these results demonstrate that PTCs have a restricted HIV reservoir, especially within longer-lived cellular subsets, which may contribute to their ability to maintain HIV remission. Additional studies are needed to explore the role of viral fitness (15), clonal expansion and the integration sites of intact proviruses in HIV remission.

Primate studies have also provided insight on strategies for delaying viral rebound. In particular, early ART therapy restricts the seeding of SIV reservoirs and lead to delayed timing of viral rebound (66, 67). Similarly, early initiation of ART has been associated with a significantly increased chance of achieving post-treatment control both within CHAMP study and others (6, 19). Prior studies of early ART treatment have found that it is effective in dramatically reducing the size of the HIV reservoir (68–71). In addition, early ART may preserve HIV-specific T cell responses (72–74). However, the VISCONTI study and others have shown that HIV-specific CD8 T cell responses in PTCs are weak compared to either SCs or viremic individuals (7, 75, 76). These results are consistent with reports that pre-ART viral loads are generally quite high in PTCs (6, 7) and that they do not tend to harbor protective HLA alleles (7, 38, 59, 75). However, other studies have not found significant differences in T cell responses between PTCs and SCs (15). In addition, there are reports from the VISCONTI study that early HIV treatment in PTCs preserves robust poly-functional CD4+ responses to HIV (77). Finally, there have been several reports that early ART initiation in infants may also lead to long-term HIV remission (76, 78, 79). In the first reported case, known as the “Mississippi baby,” the infant initiated ART 30 h after delivery until 18 months of age. ART remission was achieved without detectable HIV-specific antibody or T cell responses (78), but viral rebound occurred ~2 years after ART discontinuation (80). In the second case, the infant became infected despite 6 weeks of Zidovudine prophylaxis after delivery and initiated ART at 3 months of age. ART was discontinued between 5 and 7 years of age and viral control has been documented for ~12 years despite several transient viral blips, a detectable replication-competent reservoir, and weak HIV-specific CD8+ T cell responses (76). The final report is that of a child who initiated 40 weeks of ART at day 61 after delivery as part of the Children with HIV Early antiretroviral therapy (CHER) trial (81, 82). The child has maintained viral suppression for almost 9 years after ART discontinuation, with detectable HIV DNA and residual viremia, low level of HIV-specific antibody and weak T cell response (79). Importantly, none of these children harbored the protective HLA class I alleles B^*^27 or B^*^57 associated with spontaneous viral control and levels of immune activation during HIV remission were low in all three children (76, 78, 79). These cases also highlight that post-treatment control in children can occur with a range of ART initiation times (between 30 h and 2–3 months after delivery), HIV subtypes (B, H, and C in the three cases, respectively), and duration of ART (10 months to 6 years) (76, 78, 79). Although these studies support the possibility of HIV remission in early-treated children, the frequency of post-treatment control appears to be rare as only 1 of 227 children in the CHER trial achieved this outcome (79) and smaller studies of treatment interruption in children have failed to detect any PTCs (83).

Early ART initiation has also been shown to preserve HIV-specific humoral immunity by preserving memory B cell numbers and function (84, 85). There are reports from a small case series that PTCs may harbor high levels of autologous neutralizing antibodies (15), although that has not been replicated in other studies (8, 75).

## Knowledge Gaps and Unanswered Questions

Among the top priorities of the HIV field is the search for therapeutic interventions that can lead to sustained ART-free HIV remission (41). Understanding the mechanisms and predictors of post-treatment control would represent a key step toward that goal as PTCs represent a realistic model for the functional cure of HIV infection. Only in the past few years have interest heightened in the study of PTCs and a host of important questions remain unanswered. First, it has become clear that early initiation of ART is not only associated with personal health and public health benefits but may also lower the barrier to HIV remission and post-treatment control. However, the optimal timing of ART during early HIV infection is unknown. It is interesting to note that the vast majority of PTCs in the VISCONTI and CHAMP studies initiated ART during Fiebig stages III-V (6, 7) and that a small treatment interruption study of individuals who initiated ART during Fiebig I did not identify any PTCs as all individuals demonstrated rapid viral rebound (86). While extremely early initiation of ART will limit the extent of HIV reservoir seeding (87), additional research is needed to assess whether a slight delay in ART initiation allows for the further maturation of the HIV-specific immune response that may be important for post-treatment control.

As noted above, there is increasing evidence that PTCs do not appear to mediate HIV suppression through the same CTL and HLA-mediated mechanisms as SCs. While important, the favorable genetic profiles of SCs have not been easily translatable to therapeutics and the elucidation of the mechanisms of control in PTCs may have a greater impact on the design and evaluation of the next generation of HIV therapeutics. Studies of the HIV reservoir in PTCs have revealed the restricted size of the reservoir, including the intact proviral genomes (64). This, however, does not fully explain post-treatment control, especially given our experience in hematopoietic stem cell transplant participants who can dramatically lower their peripheral reservoir size, but are unable to maintain HIV remission (88). Additional studies are needed to assess potential differences in the distribution of infected cell types (7), cellular transcription environment, integration sites, and other factors that could contribute to the maintenance of a “deeper” state of viral latency (89).

Finally, little is known about the clinical implications of post-treatment control. While SCs can maintain low or undetectable viremia in the absence of ART, the ongoing viral replication and immune response in SCs may be associated with adverse consequences, including the progressive loss of CD4+ T cells in some individuals, increased T cell activation and inflammation (90–93). Chronic immune activation and systemic inflammation has been associated with poor clinical outcomes in non-controllers (94–97) but also in SCs, who are reported to have an increased risk of cardiovascular disease (98) and hospitalization (58), although the extent of this risk is still a matter of some uncertainty (99, 100). There is some evidence that PTCs may not exhibit the same heightened levels of immune activation as SCs (7, 10), but additional studies are needed to confirm these findings and to assess the long-term clinical implications of sustained HIV remission.

## Author Contributions

All authors listed have made a substantial, direct and intellectual contribution to the work, and approved it for publication.

### Conflict of Interest Statement

The authors declare that the research was conducted in the absence of any commercial or financial relationships that could be construed as a potential conflict of interest.
