# Supplementary material for: Learning From the Exceptions: HIV Remission in Post-treatment Controllers
Source: Front Immunol. 2019 Jul 24;10:1749. doi: 10.3389/fimmu.2019.01749 (PMC6668499; doi:10.3389/fimmu.2019.01749)
Supplement: Supplementary file 1 [file Table_1.pdf]

Supplementary Table S1. The summary of post-treatment controllers (PTCs) and viral control failure after treatment interruption (TI) definitions.

Study	Post-treatment control definition	Viral control failure definition
Hocqueloux, 2010 **(9)**	Viral load <50 copies/ml for at least 24 months post TI	2 consecutive viral rebound with viral load >50 copies/ml in 24 months post TI
Goujard, 2012 **(10)**	Viral load <50 copies/ml for more than 12 months post TI	2 consecutive viral rebound with viral load >50 copies/ml in at least 12 months post TI
Lodi, 2012 **(11)**	Viral load <50 copies/ml for in 24 months post TI	2 consecutive viral rebound with viral load >50 copies/ml in 24 months post TI
Saez-Cirion, 2013 **(7)**	Viral load <400 copies/ml for at least 24 months post TI	4 consecutive viral rebound with viral load >400 copies/ml in 24 months post TI
Stohr, 2013 **(12)**	Viral load <400 copies/ml for 12 weeks post TI	1 viral rebound with viral load >400 copies/ml in 12 weeks post TI
Van Gulk, 2012 **(15)**	Viral load <50 copies/ml for 6 months post TI	N/A
Assoumou, 2015 **(16)**	Viral load <400 copies/ml for 12 months post TI	2 consecutive or 1 plus treatment resumption viral rebound with viral load >400 copies/ml in 12 months post TI
Calin, 2016 **(17)**	Viral load <400 copies/ml for 56 weeks post TI	1 viral rebound with viral load >400 copies/ml in 56 weeks post TI
Perkins, 2017 **(18)**	Viral load <400 copies/ml for 6 months to 2 years post TI in transient controllers and more than 2 years for durable controllers	1 viral rebound with viral load >400 copies/ml (not specified exactly in how long post TI)
Fidler, 2017 **(19)**	Viral load <50 copies/ml for at least 24 months post TI	2 consecutive HIV-1 RNA viral load >50 copies/ml
Namazi, 2018 **(6)***	Viral load ≤400 copies/ml for two thirds or more of time points in at least 24 weeks post TI	Viral load >400 copies/ml were acceptable if the participant was subsequently able to suppress to ≤400 copies/ml in 24 weeks post TI

*The CHAMP study includes participants from 8 AIDS Clinical Trials Group (ACTG) studies (ACTG 371 (20), A5024 (21), A5068 (22), A5102 (23), A5130 (24), A5170 (25), A5187 (26), and A5197 (27)), the Montreal Primary HIV Infection Cohort (Montreal PIC) (28), the Seattle Primary Infection Program (SeaPIP) (13,29), the University of California San Diego Primary Infection Cohort (UCSD PIC) (14), a National Institutes of Health (NIH) therapeutic vaccine trial (31), the University of California San Francisco (UCSF) OPTIONS study (31), and the Ragon HIV Controllers cohort (32).
